# Supplementary figures and images for: Canine bocavirus-2 infection and its possible association with encephalopathy in domestic dogs
Source: PLoS One. 2021 Aug 12;16(8):e0255425. doi: 10.1371/journal.pone.0255425 (PMC8360608; doi:10.1371/journal.pone.0255425)

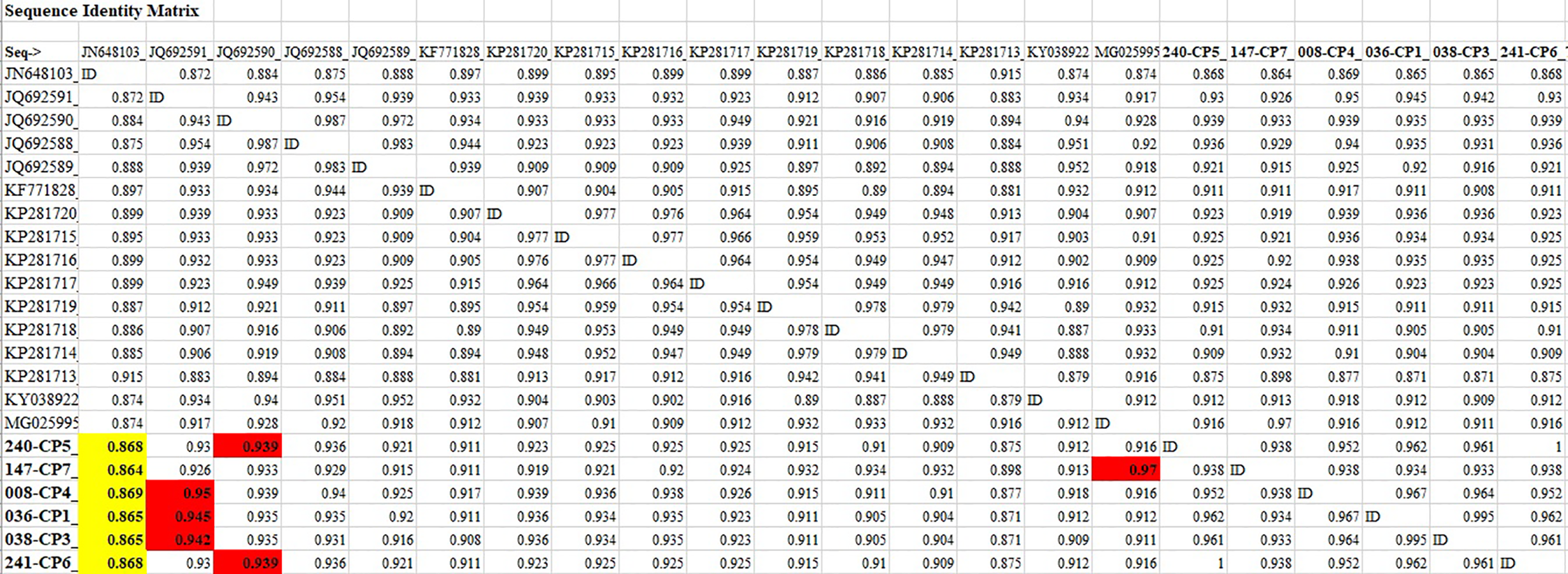

Supplement: S1 Fig — The highest and lowest nucleotide identity of obtained CBoV-2 are highlighted with red and yellow colors, respectively. (TIF) [file pone.0255425.s001.tif]

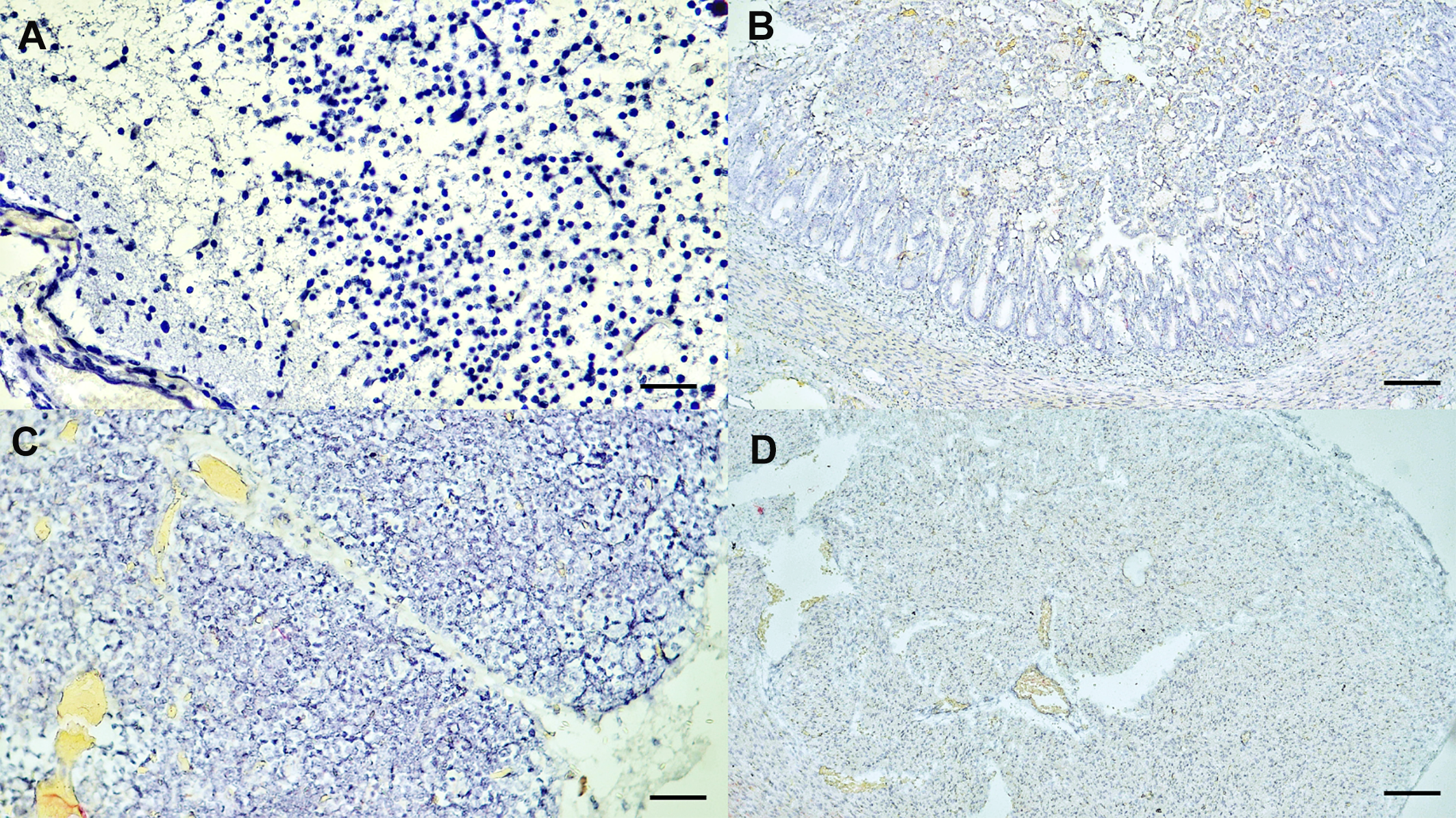

Supplement: S2 Fig — Photomicrographs of dog no. 240CP5. In situ hybridization with feline panleukopenia probe incubation served as negative controls of (A) brain, (B) intestine, (C) thymus, and (D) heart sections. Bars indicate 25 μm for (A) and 120 μm for (B-D). (TIF) [file pone.0255425.s002.tif]
