# Supplementary material for: Canine bocavirus-2 infection and its possible association with encephalopathy in domestic dogs
Source: PLoS One. 2021 Aug 12;16(8):e0255425. doi: 10.1371/journal.pone.0255425 (PMC8360608; doi:10.1371/journal.pone.0255425)
Supplement: S1 Table — The primers were designed based on alignment of multiple CBoV-2 genomes available in GenBank. (DOCX) [file pone.0255425.s003.docx]

**Canine Bocavirus-2 infection and its possible association with encephalopathy in domestic dogs**

**S1 Table. The CBoV-2-specific primers used for the full-length genome sequencing and for *in situ* hybridization (ISH).** The primers were designed based on alignment of multiple CBoV-2 genomes available in GenBank.

| Primer name | Sequence (5’-3’) |
| --- | --- |
| CBoV2_P1-F | CTWGTGGCCTGTTAATGTAT |
| CBoV2_P1-R | CCGAGATTTGTACCATCCTGC |
| CBoV2_P2-F | CTCACTTTGCCTGYTCTGGT |
| CBoV2_P2-R | GTACA GTC GTGATTGGTAC |
| CBoV2_P3-F | ACCACTACTATGGTTCACGCT |
| CBoV2_P3-R | TTGATTGAAGACCTCCATCGG |
| CBoV2_P4-F | AGGTCTTCAATCAACACCGC |
| CBoV2_P4-R | CCCTTTGTATCCGCGAGA |
| CBoV2_P5-F | TCTCGCGGATACAAAGG |
| CBoV2_P5-R | GTGTAGGAAAGTATCGTCTG |
| CBoV2_P6-F | CCAGACGATACTTTCCTAC |
| CBoV2_P6-R | CCGTTTAGTGGGCATG |
| CBoV-ISHNS-F^*^ | CTTCAGATGGTTCACATTAAGAT |
| CBoV-ISHNS-R^*^ | GGCAGYAGCTGAGAGTCTTT |
| CBoV-ISHVP-F^¥^ | TCTAATGATGTTGTTATGCA |
| CBoV-ISHVP-R^¥^ | CATCGAGATAGGACTGATA |

^*^Primer used for ISH probe construction targeting VP gene of CBoV-2

**^¥^** Primers used for ISH probe construction targeting NS gene of CBoV
