# Supplementary material for: Canine bocavirus-2 infection and its possible association with encephalopathy in domestic dogs
Source: PLoS One. 2021 Aug 12;16(8):e0255425. doi: 10.1371/journal.pone.0255425 (PMC8360608; doi:10.1371/journal.pone.0255425)
Supplement: S2 Table — The CBoV-2 genomes were detected using conventional polymerase chain reaction (PCR) and in situ hybridization (ISH). (DOCX) [file pone.0255425.s004.docx]

**Canine Bocavirus-2 infection and its possible association with encephalopathy in domestic dogs**

**S2 Table**. **Detection of CBoV-2 in various organs of selected dogs.** The CBoV-2 genomes were detected using conventional polymerase chain reaction (PCR) and *in situ* hybridization (ISH).

| **Animal** | **Sample** | **Conventional PCR** | **ISH** |
| --- | --- | --- | --- |
| **036CP1** | Heart | (-) | (-) |
|  | Liver | (+) | (-) |
|  | Brain | (+) | N/A |
|  | Mesenteric Ln | (+) | N/A |
|  | Kidneys | (+) | (-) |
|  | Intestines | (+) | (+) strong |
|  | Lung | (+) | (-) |
|  | Spleen | (-) | (-) |
|  | Thymus | (+) | (+) weak |
|  | Trachea | (+) | N/A |
| **037CP2** | Heart | (+) | (+) weak |
|  | Liver | (+) | (-) |
|  | Brain | (+) | (+) strong |
|  | Mesenteric Ln | (-) | (-) |
|  | Kidneys | (+) | N/A |
|  | Intestines | (+) | (+) strong |
|  | Lung | (+) | (-) |
|  | Spleen | (+) | (-) |
|  | Thymus | (+) | (+) strong |
|  | Trachea | (+) | (-) |
| **038CP3** | Heart | (+) | (+) strong |
|  | Liver | (+) | (-) |
|  | Brain | (+) | (+) strong |
|  | Mesenteric Ln | (+) | (+) strong |
|  | Kidneys | (+) | N/A |
|  | Intestines | (+) | (+) strong |
|  | Lung | (-) | (-) |
|  | Spleen | (+) | (+) weak |
|  | Thymus | (+) | (+) weak |
|  | Trachea | (+) | N/A |
| **008CP4** | Heart | (+) | (+) weak |
|  | Liver | (+) | N/A |
|  | Brain | (+) | (+) weak |
|  | Mesenteric Ln | (+) | (+) strong |
|  | Kidneys | (+) | N/A |
|  | Intestines | (+) | (+) strong |
|  | Lung | (+) | N/A |
|  | Spleen | (+) | N/A |
|  | Thymus | (+) | (+) strong |
|  | Trachea | (-) | (-) |
| **240CP5** | Heart | (-) | (+) weak |
|  | Liver | (+) | (-) |
|  | Brain | (-) | (+) strong |
|  | Mesenteric Ln | (-) | N/A |
|  | Kidneys | (-) | (-) |
|  | Intestines | (+) | N/A |
|  | Lung | (+) | N/A |
|  | Spleen | (-) | N/A |
|  | Thymus | (-) | N/A |
|  | Trachea | (+) | N/A |
| **241CP6** | Heart | (+) | N/A |
|  | Liver | (-) | N/A |
|  | Brain | (+) | (+) strong |
|  | Mesenteric Ln | (+) | N/A |
|  | Kidneys | (-) | N/A |
|  | Intestines | (+) | (+) strong |
|  | Lung | (+) | N/A |
|  | Spleen | (-) | N/A |
|  | Thymus | (+) | N/A |
|  | Trachea | (-) | N/A |
| **147CP7** | Heart | (+) | N/A |
|  | Liver | (+) | N/A |
|  | Brain | (+) | (-) |
|  | Mesenteric Ln | (+) | (+) weak |
|  | Kidneys | (-) | N/A |
|  | Intestines | (+) | (+) strong |
|  | Lung | (-) | N/A |
|  | Spleen | (+) | N/A |
|  | Thymus | (+) | N/A |
|  | Trachea | (-) | N/A |
| **065CP8** | Heart | (+) | (+) strong |
|  | Liver | (+) | N/A |
|  | Brain | (+) | N/A |
|  | Mesenteric Ln | (+) | N/A |
|  | Kidneys | (+) | N/A |
|  | Intestines | (+) | N/A |
|  | Lung | (-) | N/A |
|  | Spleen | (+) | N/A |
|  | Thymus | (+) | N/A |
|  | Trachea | (-) | N/A |
| **066CP9** | Heart | (+) | N/A |
|  | Liver | (+) | N/A |
|  | Brain | (+) | N/A |
|  | Mesenteric Ln | (+) | N/A |
|  | Kidneys | (+) | N/A |
|  | Intestines | (+) | N/A |
|  | Lung | (-) | N/A |
|  | Spleen | (-) | N/A |
|  | Thymus | (+) | N/A |
|  | Trachea | (+) | N/A |
| **067CP10** | Heart | (+) | N/A |
|  | Liver | (+) | N/A |
|  | Brain | (+) | (-) |
|  | Mesenteric Ln | (+) | N/A |
|  | Kidneys | (+) | N/A |
|  | Intestines | (+) | N/A |
|  | Lung | (+) | N/A |
|  | Spleen | (-) | N/A |
|  | Thymus | (+) | N/A |
|  | Trachea | (+) | N/A |

Mesenteric Ln: Mesenteric lymph node; (+): positive; (-): negative; N/A: not performed; strong: presented ISH signal in over 50% positive cells; weak: presented ISH signal in less than 50% positive cells.
